# Helicobacter pylori and acne vulgaris: is there a relationship?

**DOI:** 10.1007/s00403-024-03300-w

**Published:** 2024-09-14

**Authors:** Ahmed Abdelfattah Afify, Hanan Mohamed Ahmed Saleh, Abeer Farrag Hussein

**Affiliations:** Dermatology, venereology and andrology department, Faculty of medicine, Ain Shams University, Cairo, Egypt

**Keywords:** Helicobacter-pylori-acne-vulgaris-relationship

## Abstract

Background: Helicobacter pylori (H. pylori) is a gastric Gram-negative, spiral-shaped microaerophilic pathogen. H. pylori may play a potential pathogenic role in extra-intestinal diseases such as hepatobiliary, respiratory, and dermatological disorders. The latter included chronic urticaria, psoriasis and rosacea. The first report in literature on the relationship between H. pylori and acne vulgaris (AV), found association between severe AV and H. pylori infection. There are very limited data in AV patients addressing the impact of H. pylori infection on various severities. In this context, the aim of the present work was to determine the association of H. Pylori infection among AV patients and correlate it with the disease severity. Methods: This case-control study included 45 Patients with AV and 45 age and sex matched healthy volunteers as a control group. H. pylori antigen in stool and serum H. pylori antibody IgG using commercially available ELISA kits was tested in all included subjects. Results: The percentage of participants with a positive H. pylori antigen in stool and positive H. pylori antibody in serum in the whole study population was 35/90 (38. 9%) and 41/90 (45. 6%). On comparing between the percentages of positive H. pylori antigen in stool and positive H. pylori antibody in serum between the patients with AV and healthy controls, a highly statistically significant difference was found between the two groups (*P* < 0.001, *P* = 0.006). On comparing between the percentages of positive H. pylori antigen in stool and positive H. pylori antibody in serum in the patients with different grades of acne severity and healthy controls, the rate of positive H. pylori antigen in stool and positive H. pylori Ab in serum was significantly associated with severity of acne comparing with healthy controls (*p* < 0. 001). Conclusion: The rate of H. pylori infection in patients with AV is high so it may influence the pathogenesis of this skin disease. Patients with severe AV had higher rates of H. pylori antigen in stool and H. pylori antibody in serum as compared to the patients with mild AV and healthy controls.

## Introduction

Acne vulgaris (AV), is one of the most common dermatologic complaints [1]. About 85% of adolescent population and 3% of the adults between the age of 35–44 years have this disease worldwide [2].

It is a chronic inflammatory disorder of the pilosebaceous unit. The pathogenesis of AV includes disturbed sebaceous gland activity associated with hyperseborrhoea and alterations in sebum fatty acid composition, dysregulation of the hormone microenvironment, interaction with neuropeptides, follicular hyperkeratinization, Propionibacterium acnes (P. acnes), induction of inflammation and dysfunction of the innate and adaptive immunity [3]. Acne can be manifested in both inflammatory and noninflammatory forms [4].

Helicobacter pylori (H. pylori) is a gastric Gram-negative, spiral-shaped microaerophilic pathogen closely associated with gastric and extra-gastric diseases [5, 6]. Appropriately half of the global population is infected with H. pylori, and the prevalence of H. pylori varied among regions, which appeared to be explained by the differences in economic and social conditions [7].

The most common routes of H. pylori transmission are oral-to-oral and fecal-to-oral routes. The colonization of H. pylori itself does not cause any symptoms, and < 20% of all infected patients will develop symptoms following infection [8].

It has been shown that H. pylori may play a potential pathogenic role in extra-intestinal diseases such as hepatobiliary, respiratory, cardiovascular, and dermatological disorders. The latter included chronic urticaria, psoriasis, henoch-schonlein purpura, rosacea, behcet’s disease, alopecia areata and sweet’s syndrome [9].

The first report in the literature on the relationship between H. pylori and AV, found that there is association between severe AV and H. pylori infection [10].

Khalid et al. (2021) suggested that AV and H. pylori infection may be related. However, there is insignificant evidence to support a casual association between H. pylori infection and AV per se. More so, there are very limited data in AV patients addressing the impact of H. pylori infection on various severities [11].

In this context, the aim of the present work was to determine the association of H. Pylori infection among AV patients and correlate it with the disease severity.

## Patients and methods

### Participants

This case-control study included 45 Patients with AV and 45 age and sex matched healthy volunteers as a control group. They were recruited from the Dermatology outpatient clinic, Ain Shams University Hospitals and Teba specialized hospital in Luxur government. The study was done in the period from November 2021 to October 2022. Patients < 12 years and > 40 years, patients with a history of drug intake that affects H. pylori such as proton pump inhibitors, clarithromycin, tetracyclines, amoxicillin or metronidazole within the previous 1 month and patients who received any topical or systemic treatment for AV in the past 3 months were excluded. After the approval of Ethics Committee of Faculty of Medicine, Ain Shams University (Approval number: FMASU 670 / 2021), all participants signed an informed consent after explaining for them the objective of the study.

### Methods

I- Full history taking including personal history, drug history, family history and history of the present illness with special emphasis on: Onset, course and duration of the disease.

II- Clinical examination:

(a) General examination. (b) Dermatological examination: The AV patients were examined for the type of acne (Comedonal, inflammatory or mixed) and the site of AV (Forehead, cheeks, nose, chin, arms, chest and upper back).

III– Severity indices:

For evaluation of severity of the disease, patients with AV were classified into mild, moderate, severe according to the global acne grading system (GAGS). The GAGS score ranges from 0 (no acne), 1–18 (mild acne), 19–30 (moderate acne), 31–38 (severe acne), and > or = 39 (very severe acne) [12].

lV- Laboratory investigations:

H. pylori antigen in stool (catalogue no. HPY35-k01, Eagle Biosciences) and serum H. pylori antibody IgG (Catalog No: KA0219, Abnova company) using commercially available ELISA kits following the manufacturer guidelines.

### Sample collection, handling and storage

#### Stool specimen collection, handling and storage

Fresh stool samples were collected from each participant in the study and control group into stool sample collection containers. A minimum of 1–2 mL liquid stool sample or 1–2 g solid sample was the needed sample. The collected fecal sample was transported to the lab in a frozen condition (-20 °C). If the stool sample was collected and tested the same day, it is allowed to be stored at 2–8 °C.

#### Blood specimen collection, handling and storage

Blood (the volume of blood needed 1 milliliter) samples were taken from each participant in the study and control group and was directly transferred to laboratory for detection of H. pylori immunoglobulin G (Ig G) antibody in serum. Serum prepared from a whole blood specimen obtained by acceptable medical techniques. This kit is for use with serum samples without additives only. Specimens may be refrigerated at 2–8 C^o^ for up to 7 days.

#### Assay procedure

All samples and standards were prepared as instructed.

#### H. pylori Ag in stool was measured with monoclonal antibodies-based ELISA kit by sandwich technique by the following steps

A sufficient number of H. Pylori monoclonal antibody-coated microwell strips were placed in a frame to run H. Pylori negative control (1x Assay buffer), positive control and unknown samples induplicate. 100µL of controls (use1x Assay buffer as a negative control) and diluted patient stool samples were added into the designated microwell. Mix by gently tapping the plate. The plate was covered with one plate sealer. Cover with foil or other material to protect from light. The plate was incubated at room temperature for 1 h. The plate sealer was removed and aspiration of the contents of each well was done. Each well was washed 5 times by dispensing 350µL of working wash solution into each well, then complete aspiration of the contents was done. 100 µl of Stop Solution was added to each well and mixed gently. The color change was measured spectrophotometrically at a wavelength of 450 nm.

#### IgG antibodies against H. pylori in human serum was assessed with ELISA by the following steps

The desired number of coated wells were secured in the holder. 1:40 dilution of test samples, negative control, positive control, and calibrator was prepared by adding 5 µl of the sample to 200 µl of sample diluent. Mix well. 100 µl of diluted sera, calibrator, and controls were dispensed into the appropriate wells. For the reagent blank, 100 µl sample diluent was dispensed in 1 A well position. They were mixed well for 10 s and incubated at room temperature for 30 min. At the end of the incubation period, liquid was removed from all wells. The microtiter wells were rinsed and flicked 4 times with diluted wash buffer (1×) and then one time with distilled water. 100 µl of enzyme conjugate was dispensed to each well and mixed gently for 10 s. Incubation at room temperature for 30 min was done. Enzyme conjugate was removed from all wells. The microtiter wells were rinsed and flicked 4 times with diluted wash buffer (1×) and then one time with distilled water. 100 µl of TMB Reagent was added to each well and mixed gently for 10 s and incubated at room temperature for 20 min. 100 µl of Stop Solution was added to each well including the 2 blanks and mixed gently for 30 s (the blue color changed to yellow color at that moment). The optical density was determined at 450 nm with a microtiter plate reader.

#### Interpretation of the results

The concentration value of the patients and controls samples were determined from a calibration standard curve which was constructed by plotting the optical density of each standard with respect to the corresponding units’ values, using a linear regression equation.

### Statistical methodology

The data were analyzed using Statistical Package for Social Science (IBM Corp, released 2013. IBM SPSS statistics for windows, V. 22. 0. Armonk, NY. USA). Parametric quantitative data were expressed as mean ± standard deviation (SD). Non-Parametric quantitative data were expressed as median and IQR. Qualitative data were described as frequency and percentage.

The categorical variable was analyzed with frequency and percentage. Chi-squared test was used to evaluate the differences in categorical data. Kruskal-Wallis Test was used to evaluate the differences in nonparametric quantitative variables which was not normally distributed and involving more than two groups. Adjusted Mann-Whitney U Test was used to evaluate the differences between two nonparametric quantitative variables. Spearman correlation was used to evaluate the correlation between two variables. All P values were two-tailed and P-value ≤ 0.05 was considered statistically significant and ≤ 0.01 was considered statistically highly significant.

## Results

### Demographic data

This case-control study included 45 Patients with AV and 45 age and sex matched healthy volunteers as a control group. Sex distribution among the whole study population were 58 females (64. 4%) and 32 males (35. 6%). Age among the whole study population were from 12 to 37 years with mean age = 18.7 ± 4.4 years.

Included AV patients were 29 females (64. 4%) and 16 males (35. 6%). Age ranged from 13 to 36 years with mean age = 18.6 ± 4.5 years (Table 1).

**Table 1:** Sex and age distribution among case/control groups

	Female		Male				
	Number	Percent	Number	Percent	X^2^	*P* value	Sig
**Case group**	29	64. 4%	16	35. 6%	0	1	NS
**Control group**	29	64. 4%	16	35. 6%			
**Age distribution**							
	**Mean ± SD**	**Range**	**Interquartile Range**	Test value*	**P value**		**Sig**
**Case group**	18.6 ± 4.5	13–36	5	0.114	0.886		NS
**Control group**	18.7 ± 4.3	12–37	4				

X2 = Chi-Square Test, NS = non-significant, *= Independent samples Test

Included healthy individuals were 29 females (64.4%) and 16 males (35.6%). Age ranged from 12 to 37 years with mean age = 18.7 ± 4.3 years (Table 1). There was no statistically significant difference in age and sex between the two groups (*P* = 0.743, *P* = 1) (Table 1).

### Clinical data

Duration of AV range from 7 to 144 months with a median (36 months). Family history of AV was positive in 39 patients (86.6%). The type of AV was inflammatory in 12 patients (26. 7%), comedonal in 6 patients (13.3%) and mixed in 27 patients (60. 0%).

Involved acne sites were (forehead & cheeks) in 13 patients (28. 9%), (forehead, cheeks and nose) in 3 patients (6. 7%), (forehead, cheeks, nose and chin) in 12 patients (26. 7%), (forehead, cheeks, nose, chin, chest and upper back) in 7 patients (15. 6%), (forehead, cheeks, nose and chest) in 3 patients (6. 7%), (forehead, cheeks, nose and upper back) in 3 patients (6. 7%), (forehead, cheeks, chin, chest and upper back) in 1 patient (2. 2%), (forehead, cheeks, nose and upper back) in 1 patient (2. 2%), (forehead, cheeks and chin) in 1 patient (2. 2%) and (forehead, cheeks and upper back) in 1 patient (2. 2%).

According to the GAGS assessment, the severity of AV was classified as mild in 16/45 (35. 56%), moderate in 16/45 (35. 56%), and severe in 13/45 (28. 88%) of the patients.

### Laboratory data

The percentage of participants with a positive H. pylori antigen in stool in the whole study population was 35/90 (38. 9%). The percentage of participants with a positive H. pylori antibody in serum in the whole study population was 41/90 (45. 6%).

On comparing between the percentages of positive H. pylori antigen in stool between the patients with AV and healthy controls, a highly statistically significant difference was found between the two groups (*P* < 0.001) (Table 2).

**Table 2:** Positive Helicobacter pylori Ag in stool and positive Helicobacter pylori Ab in serum among case/ control groups

Helicobacter pylori Ag in stool	Control				Case			X^2^	P value		Sig
	Count			Percent	Count		Percent				
**Negative**	36			80%	19		42. 2%	13. 512	< 0. 001		HS
**Positive**	9			20%	26		57. 8%				
**Helicobacter pylori Ab in serum**											
**Negative**	31			68. 9%	18		40%	7. 571	0. 006		HS
**Positive**		14	31. 1%		27	60%					

X2 = Chi-Square Tests, HS = Significant

On comparing between the percentages of positive H. pylori antibody in serum between the patients with AV and healthy controls, a highly statistically significant difference was found between the two groups (*P* = 0.006) (Table 2).

On comparing between the percentages of positive H. pylori antigen in stool in the patients with different grades of acne severity and healthy controls, the rate of positive H. pylori antigen in stool was significantly associated with severity of acne comparing with healthy controls (*p* < 0. 001) (Table 3).

**Table 3:** Percentage of patients with positive Helicobacter pylori Ag in stool according to severity of Acne

	Control		Mild			Moderate			Severe			Test value *	*P* value	Sig
	Count	Percent	Count		Percent	Count		Percent	Count	Percent				
Negative	36	80%	12		75%	6		37. 5%	1	7. 7%		27. 113	< 0. 001	S
Positive	9	20%	4		25%	10		62. 5%	12	92. 3%				
**Pairwise Comparisons of Severity of Acne**														
**Sample 1-Sample 2**				**Test Statistic**			**Adjusted p value**				**Sig**			
Control-Severe				-32. 538			< 0. 001*				S			
Mild-Severe				-30. 288			. 001*				S			
Mild-Moderate				-16. 875			. 183				NS			
Control-Mild				-2. 250			1. 000				NS			
Control-Moderate				-19. 125			. 017				NS			
Moderate-Severe				-13. 413			. 621				NS			

*=Kruskal-Wallis Test, S = Significant, NS = non-significant

On comparison, the patients with severe AV had significantly higher rate of H. pylori Ag in stool (92. 3%) as compared to healthy controls (20%, *p* < 0. 001) and compared to patients with mild AV (25%, p=. 001), while no statistical significance between mild AV-moderate AV (*p* = 0. 183), healthy controls-mild AV (*p* = 1. 000), healthy controls-moderate AV (p=. 017) and moderate AV-severe AV (*p* = 0. 621) (Table 3).

On comparing between the percentages of positive H. pylori antibody in serum in the patients with different grades of acne severity and healthy controls, the rate of positive H. pylori Ab in serum was significantly associated with severity of acne comparing with healthy controls (*P* < 0. 001) (Table 4).

**Table 4:** Helicobacter pylori Ab in serum according to severity of Acne

	Control		Mild			Moderate			Severe			Test value*	*P* value	Sig
	Count	Percent	Count		Percent	Count		Percent	Count	Percent				
Negative	31	68. 9%	11		68. 8%	6		37. 5%	1	7. 7%		18.210	< 0.001	S
Positive	14	31. 1%	5		31. 3%	10		62. 5%	12	92. 3%				
**Pairwise Comparisons of Severity of Acne**														
**Sample 1-Sample 2**				**Test Statistic**			**Adjusted p value**				**Sig**			
Control-Severe				-27. 538			. 001				S			
Mild-Severe				-27. 476			. 007				S			
Control-Mild				-. 062			1. 000				NS			
Control-Moderate				-14. 125			. 188				NS			
Mild-Moderate				-14. 062			. 465				NS			
Moderate-Severe				-13. 413			. 666				NS			

*=Kruskal-Wallis Test, S = Significant, NS = Non-significant

On comparison, the patients with severe AV had significantly higher rate of H. pylori Ab in serum (92. 3%) as compared to healthy controls (31. 1%, *p* = 0. 001) and compared to patients with mild AV (31. 3%, p=. 007), while no statistical significance between healthy controls-mild AV (*p* = 1. 000), healthy controls-moderate (p=. 188), mild AV-moderate AV (*p* = 0. 465) and moderate AV-severe AV (*p* = 0. 666) (Table 4).

### Correlation between different variables of AV patients and H. pylori Ag in stool and ab in serum

The number of AV involved sites and severity of AV were significantly correlated with H. pylori Ag in stool and H. pylori Ab in serum (Table 8). While there was no statistically significant correlation between (the type of AV, onset of AV and duration of AV) and H. pylori Ag in stool and H. pylori Ab in serum (Table 5).

**Table 5:** Correlation between different variables of AV patients and H. Pylori Ag in stool and ab in serum

	Helicobacter pylori Ag in stool		Helicobacter pylori Ab in serum	
	ρ	*P* value	ρ	*P* value
Type of Acne	-. 012	. 938	. 024	. 876
Number of involved sites	. 414	. 005*	. 358	. 016*
Severity of Acne	. 548	< 0. 001*	. 499	< 0. 001*
Onset of Acne (y)	-. 133	. 383	-. 090	. 556
Duration of Acne (m)	-. 141	. 355	-. 074	. 630

ρ = spearman correlation, *= significant

## Discussion

Acne vulgaris is one of the most common skin diseases worldwide that significantly affects the patients’ life quality and often associated with anxiety and depression [13].

It is a chronic inflammatory skin disorder. Multiple factors contribute to acne pathogenesis, including increased sebum production, aberrant keratinization of the pilosebaceous duct, bacteria such as P. acnes, hormonal influences, the skin microbiome and chronic inflammation [14].

There is a growing concern toward the role of H. pylori in the pathogenesis of many skin diseases [9]. It was found that H. pylori infection is involved in the development of rosacea. It is suggested that rosacea patients should be tested for H. pylori infection, the H. pylori-positive rosacea patients should be treated with eradication of H. pylori, so as to enhance the therapeutic effect on rosacea [15].

This study was conducted to determine the prevalence of H. pylori infection in patients with AV and to correlate it with the disease severity.

In this study H. pylori antigens in stool were detected in 57. 8% of patients with AV and H. pylori (IgG) antibodies in serum were detected in 60% of patients with AV and these were significantly higher from the rates in healthy controls. Also, when the patients were stratified according to the severity of acne, the group presented with severe AV had significantly higher rate of positive H. pylori antigen in stool and positive H. pylori antibody in serum as compared to the healthy controls and mild AV patients. However, the differences between healthy controls and patients with mild or moderate AV were not statistically significant. The above-mentioned findings agreed with the observations of Khodaeinai et al., (2014) the first report in the literature on the relationship between H. pylori and AV, showed in his case-control study on 100 (25 control, 75 cases) individuals. The rate of H. pylori infection was found in 56% of control group, 60% in the cases of mild AV, 72% of moderate and 88% of severe AV patients. Frequency of H. pylori infection was significantly associated with severity of acne comparing with controls. Mean serum IgG was also significantly high in the group with severe disease [10].

Also, our findings agreed with the findings in Saleh et al., (2020) which showed that the patients with severe AV had significantly higher levels of fecal H. pylori antigen as compared to the patients with mild AV, moderate AV, and healthy controls (P <. 001) [16]. Also, Khalid et al., (2021) in his cross-sectional study which included 135 patients, 48 (35.56%) females and 87 (64.44%) males with male to female ratio of 1.8:1.0. The frequency of H. pylori infection in patients of AV was seen in 107 (79.26%) patients [11].

A prospective cohort study was done by Khashaba et al., (2020) to detect the prevalence of H. pylori in AV patients of different severities and the impact of its eradication on clinical outcome. It included 66 patients along with 22 controls. There was statistically significant increase in anti-Helicobacter immunoglobulin G index in severe acne group and statistically significant decrease in total lesion count after triple therapy in all groups (*P* = 0.002, 0.04, 0.001) [17].

In our study the number of AV involved sites and severity of AV were positively correlated with the rates of H. pylori antigen in stool and H. pylori antibody in serum. The observations of our study indicate that the prevalence rate of H. pylori infection in patients of AV is high and H. pylori infection may be related to increasing the severity of AV.

A possible mechanism for the association of AV and H. pylori infection may be related to that H. pylori leads to formation of increased number of reactive oxygen species like inflammatory cytokines and superoxide. These toxic metabolites enhance the inflammation of the gastric mucosa as well as pilosebaceous units in skin leading to AV [16].

Secondly, there is a mechanism suggested that H. pylori infection might significantly be associated with seborrhea. Another possible mechanism which can lead to the development of AV is the direct involvement of H. pylori. According to researches, there may be cross-mimicry between H. pylori and extra digestive antigens present in the skin. Further H. pylori produces an enzyme, lipase. This enzyme has significant role in the pathogenesis of AV. This lipase activity of H. pylori significantly correlates the association of H. pylori with AV [11].

Additionally, the antibiotics that are known to be effective in AV such as metronidazole, tetracycline, and doxycycline, are also effective against H. pylori infection. Such observation may also suggest a possible role of H. pylori infection, in the pathogenesis of AV [18].

So, we conclude that the rate of H. pylori infection in patients with AV is high so it may influence the pathogenesis of this skin disease. Patients with severe AV had higher rates of H. pylori antigen in stool and H. pylori antibody in serum as compared to the patients with mild AV and healthy controls.

## Data Availability

No datasets were generated or analysed during the current study.
